# Cognitive profile and frailty in patients with idiopathic normal pressure hydrocephalus

**DOI:** 10.3389/fneur.2025.1644507

**Published:** 2025-09-04

**Authors:** Magnhild S. Dejgaard, Per Kristian Eide, Gro Gujord Tangen, Eva Skovlund, Geir Selbæk, Torgeir Bruun Wyller

**Affiliations:** ^1^Department of Geriatric Medicine, Oslo University Hospital, Oslo, Norway; ^2^Institute of Clinical Medicine, University of Oslo, Oslo, Norway; ^3^Department of Neurosurgery, Oslo University Hospital, Oslo, Norway; ^4^KG Jebsen Centre for Brain Fluid Research, Oslo, Norway; ^5^Norwegian National Center for Ageing and Health, Vestfold Hospital Trust, Tønsberg, Norway; ^6^Department of Public Health and Nursing, Norwegian University of Science and Technology (NTNU), Trondheim, Norway

**Keywords:** normal pressure hydrocephalus, gait disorder, dementia, frailty, mild cognitive impairment

## Abstract

**Background:**

Cognition and frailty are sparsely studied in patients with idiopathic normal pressure hydrocephalus (iNPH). We aimed to describe the preoperative cognitive function compared with normative data and frailty profile in iNPH patients accepted for shunt surgery.

**Methods:**

All patients were diagnosed according to international guidelines and underwent a standardized cognitive and physical examination and a geriatric assessment prior to surgery. *Z*-scores for the cognitive tests were calculated based on age and education adjusted population norms.

**Results:**

The study cohort included 276 iNPH patients accepted for shunt surgery. Mean ± SD age was 73.1 ± 5.7 years, education 12.5 ± 3.8 years, and 61% were male. The median (IQR) score on the Mini-Mental State Evaluation was 27 (24–29), and the median (IQR) Clock Drawing test score was 4 (3–5). Mean (SD) *z*-score for immediate verbal recall was −1.74 (0.98), for delayed recall −1.66 (1.01), for figure copying −0.85 (1.35) for Trail Making Test A -1.50 (1.09), for Trail Making Test B −1.88 (1.03), for phonemic fluency −1.46 (1.10), and for semantic fluency −1.59 (1.20). Cluster analysis identified three groups, mainly differing regarding visuospatial function. The mean (SD) Frailty Index score was 0.23 (0.13), indicating mild frailty. The frailty domain most affected was physical function.

**Conclusion:**

iNPH patients showed reduced cognitive function across all domains. The patient group is rather heterogeneous regarding cognitive symptoms, and no specific cognitive profile was identified. Cognitive assessment offers limited utility for diagnosing a typical pattern specific for iNPH but is important due to the complex needs for this patient group. Whether cognitive and frailty profile can be used to identify shunt responders, must be assessed in longitudinal studies.

## Introduction

Idiopathic normal pressure hydrocephalus (iNPH) is characterized by one or more of the symptoms gait disturbance, cognitive decline and urinary incontinence (1, 2). While the patterns of gait disturbances have been well characterized (3), our understanding of the cognitive profile specific for iNPH remains vaguer even though cognitive impairment is frequently present. Selection for surgery is challenging, especially when cognitive symptoms are suggestive for other neurodegenerative diseases, such as vascular dementia or Alzheimer disease. Neuroimaging improves patient selection, but Alzheimer and cerebrovascular findings are often present, co-pathology is common, and the diseases overlaps. The cognitive profile in iNPH is often described as subcortical with impaired attention, reduced psychomotor speed and reduced memory (4) which is similar to the profile of vascular dementia (VD), making the conditions difficult to separate. There is a need to better characterize the cognitive profile of iNPH patients given recent reports of markedly higher prevalence of iNPH than previously anticipated (5, 6); a recent report estimated a prevalence of iNPH to 1.5% among people aged 70 years (7). Moreover, iNPH is a progressive disease with a gradual clinical deterioration and increased risk for death (8, 9). Frailty is emerging as an important risk factor for mortality and postoperative complications but has to a limited degree been studied in iNPH (10). The aim of this study was to characterize the cognitive profile compared with normative data, and further to assess the degree and profile of frailty in iNPH patients.

## Methods

### Study population

**Figure 1:**
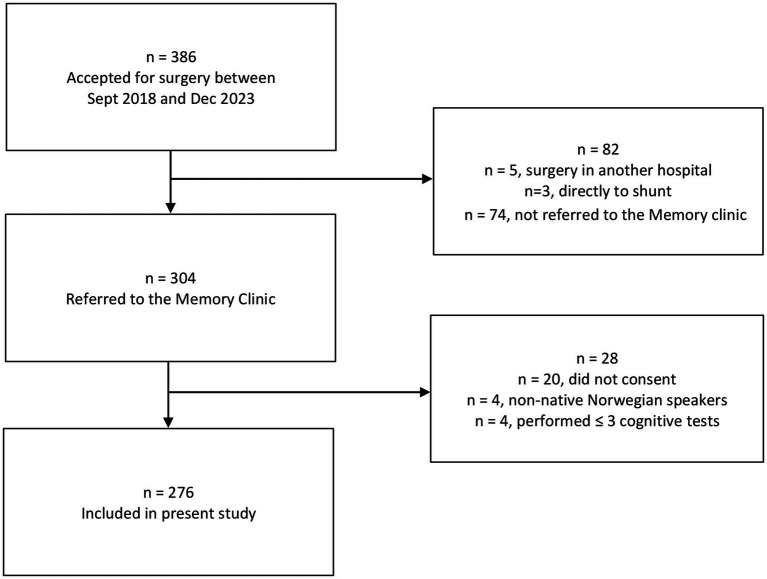
Flow chart for patients and reasons for dropout.

This is a cross-sectional study, including patients from Oslo University Hospital, Rikshospitalet with iNPH, accepted for shunt surgery between September 2018 and December 2023. All patients met the diagnostic criteria for possible iNPH according to the American-European guidelines (2). Patients were selected for shunt surgery based on clinical presentation and MRI findings, supplemented either with measurements of intracranial pulse pressure waves or MRI with intrathecal gadobutrol contrast (11, 12). In the study period, patients accepted for shunt surgery were referred to the Memory Clinic at the Department of Geriatric Medicine for a standardized preoperative assessment. Non-native speakers of Norwegian and patients who had completed ≤ 3 cognitive tests were excluded from further analysis. Figure 1 shows participant flow. Participants were also included in The Norwegian Register of Persons Assessed for Cognitive Symptoms (NorCog) (13).

### Clinical assessment

Patients underwent a standardized cognitive examination by experienced geriatricians, psychiatrists or neurologists, and physical examination by skilled physiotherapists. Typically, there were 2–4 weeks between acceptance for surgery and the assessment in the Memory Clinic, and a similar period from assessment to surgery, though delays could occur due to acute diseases, etc.

### Assessment of cognitive function

We used the same standardized cognitive test battery as in NorCog, with the addition of the Rey Auditory Learning Test (RAVLT) for some of the participants. *Z*-scores were calculated using age and education-adjusted norms, indicating the number of standard deviations an individual’s result deviates from the population mean. A *z*-score of 0 denotes a result that is average compared to the normative group, a positive *z*-score indicates a performance above average, and a negative *z*-score a performance below average. The lowest possible *z*-score was set to −3 and indicates −3 SD or more from the mean. *Z*-scores were based on Norwegian normative populations whenever available.

Norwegian Revised Mini-Mental State Examination (MMSE-NR3) was scored 0–30 (higher score indicating better function). This is a cognitive screening test assessing elements of orientation, immediate and delayed recall, calculation, language, praxis, and visuoconstruction (14).

Norwegian Revised Clock Drawing Test is a cognitive screening test sensitive for spatial orientation, attention, executive function, and semantic memory and is scored 0–5 as described by Shulman (15).

The Rey Auditory Learning Test (RAVLT) and the 10-word Test from the Consortium to Establish a Registry for Alzheimer’s disease (CERAD) were used interchangeably (16, 17). Patients with a total MMSE-NR3 score below 20 or unable to recall any of the three words in MMSE-NR3 were given the 10-word test, otherwise, RAVLT was performed. Both tests evaluate immediate and delayed verbal recall. The 10-word test involves 10 words read three times, while RAVLT consists of 15 words read five times plus a distraction list. For both tests, we calculated *z*-scores for immediate and delayed recall, enabling us to present common, adjusted measures of these aspects of verbal memory (18–20). For patients aged 70–90 years and performing the 10-word test, we calculated *z*-scores using Wagle’s normative data (19). Eleven patients who completed the 10-word test were ≤ 69 years, and for these patients, Kirsebom’s norms were used (20). For the RAVLT we used Espenes’ norms from a sample of Norwegian and Swedish adults aged 49–79 years (18). Four patients who completed the RAVLT were 80 years or more, and therefore outside the age-adjusted norms. For these patients, *z*-scores were calculated as if they were 79 years old.

The Figure Construction Test from CERAD assesses visuoconstructive abilities (17). The score ranges from 0 to 11, a higher score indicating better performance. Norwegian norms were not available, so we assessed *z*-scores based on population norms from Luck (21). Twenty-nine individuals were ≥ 80 years, and therefore outside the age-adjusted norms. *Z*-scores for these patients were calculated as if they were 79 years old. In this test, a ceiling effect exists, as in most age and education strata half of the norm material achieved a full score (21).

The Trail Making Test (TMT) has two parts, A and B. TMT A features numbers from 1 to 25 scattered randomly. The participants connect them in ascending order as quickly as possible, assessing attention and psychomotor speed (22). TMT B includes thirteen numbers and twelve letters, requiring participants to connect them in alternate order (1-A-2-B-3-C) as quickly as possible. This test is considered sensitive for executive function in addition to the same functions as TMT A. Four patients were unable to complete TMT A, and 92 were unable to complete TMT B. These individuals have been assigned z scores - 3 as the presumed weakest score. Norms are from Espenes (23).

Phonemic verbal fluency test is sensitive for language abilities and executive function (17). The individual is given 60 s to generate as many words as possible beginning with each of the letter “F,” “A” and “S.” This test *z*-scores were taken from Egeland’s Norwegian norms for patients ≤ 77 years (24). For those ≥ 78 years, Norwegian norms were not available, and we used norms from Tombaugh et al. (25).

Semantic verbal fluency test is sensitive for language abilities and memory (17). The individual is asked to generate first as many animal names as possible in 60 s and then clothes in 60 s. For patients ≤ 77 years, normative data were taken from Egeland (24). For those ≥ 78 years, valid normative *z*-scores exist for animals-naming only, and are taken form Tombaugh et al. (25).

### Overall assessment of dementia severity

The Clinical Dementia Rating Scale (CDR) was used as a global assessment of cognitive impairment. This scoring system evaluates memory, orientation, judgment and problem solving, community affairs, home and hobbies, and personal care, each domain carrying a score from 0 to 3. These domain scores are aggregated using an algorithm to calculate a global score that reflects the severity of cognitive impairment, assigning more weight to the item memory. A score of 3 points indicates severe dementia, 2 points moderate dementia, 1 point mild dementia, and 0.5 points mild cognitive impairment, whereas 0 points signify the absence of cognitive impairment (26).

### Physical assessment

Timed up and go (TUG) measures the time it takes for a patient to stand up from a chair, walk a distance of 3 meters, turn 180 degrees, walk back to the chair and sit down again (27).

Short Physical Performance Battery (SPPB) evaluates balance, gait speed and lower extremity strength. Each of these tests are scored on a scale from 0 to 4, with higher score indicating better performance, 12 is the maximum score (28).

iNPH gait and balance scales are graded with a score range from 0 to 100 and are based on gait and balance evaluation. A low score representing poorer performance and a score of 100 indicating the performance of an age-matched healthy population (29).

iNPH gait comprises the number of steps and time needed to walk 10 meters, followed by tandem walking and a 180° turn. The gait is evaluated with a scale from 1 (normal) to 8 (wheelchair bound). The patient uses their usual walking aid. The gait evaluation, number of steps, and time needed are converted according to a table to a score ranging from 0 to 100.

iNPH balance is assessed by observing the patient’s ability to maintain an upright stance on one or both legs. The scale goes from 1 to 7. One point is given if the patient can stand independently on one leg for more than 30 s, whereas seven points are given if the patient is unable to stand without assistance. The rating is converted into scores from 0 to 100.

Gait speed in m/s from the iNPH 10 m walk is also used as a separate outcome (30).

### Frailty

To grade frailty, we calculated a Frailty Index (FI) based on a comprehensive geriatric assessment (31). The original FI consisted of 48 elements, such as comorbidity, activities of daily living, physical and cognitive function, and nutritional status. For each element, 0 indicates no problems and 1 indicates problems. The points are summed and divided by the number of assessed elements, resulting in a ratio between 0 and 1. For the FI to be considered as valid, a minimum of 30 elements must be available (32). We were able to score 37 of the elements, based on the clinical examination and the medical records. We did not have data on sensory loss, involuntary weight loss, grip strength and items from the NAGI areas (a tool used to assess physical function and disability) and Rosow-Breslau scale (composite measure of mobility disability). Higher scores indicate greater degree of frailty, with a score less than 0.10 indicating a fit person, 0.10–0.19 vulnerable, 0.20–0.29 mild frailty, 0.30–0.39 moderate frailty, and a score of 0.4 or higher indicating severe frailty (33, 34). For a differentiated analysis of the various domains of frailty, we similarly calculated indexes ranging from 0 to 1 (number of problems divided by number of items evaluated) for each of the FI sub-domains cardiovascular morbidity, non-cardiovascular morbidity, personal activities in daily living (pADL), instrumental activities in daily living (iADL), cognitive function and physical function. For the domains polypharmacy (1 item) and nutritional status (2 items), we alternatively report the number and percentage of patients with any problem.

### Statistical analysis

Variables were summarized by descriptive statistics. Patients were partitioned into non-overlapping groups by k-means cluster analysis based on *z*-scores on six different cognitive tests. Differences between patient characteristics in each cluster were compared by one-way ANOVA. *p*-values < 0.05 were considered to indicate statistical significance. The analyses were conducted using STATA/SE 18.0.

### Standard protocol approvals, registrations, and patient consents

The Regional Committee for Medical and Health Research Ethics (REK) (2019/547), the Data Protection Officer at Oslo University Hospital (19/14118) and the Steering Committee for the NorCog register (08/01815) approved the study. REK also approved the linkage of data from the NorCog register with data collected for the present project (08/01815). The ability to consent was assessed for all patients included, and informed consent was obtained from all participants.

## Results

In total, 386 patients were accepted for shunt surgery during the inclusion period, and 276 (71.5%) were included. Figure 1 shows patient flow and reasons for dropout. For most patients who were not referred, this was due to practical conditions such as a long travel distance to the study center or that the patients found it difficult to attend additional appointments. Mean ± SD age of the included patients was 73.1 ± 5.7 years, 167 (61%) were male and mean ± SD length of education was 12.5 ± 3.8 years. More detailed characteristics are summarized in Table 1.

**Table 1:** Demographic characteristics, overall frailty and individual frailty indicators (*n* = 276 unless otherwise specified).

Demographic data	*N* (%)	Mean (SD) score for this domain of the Frailty Index
Age, years, mean (SD) [range]	73.1 (5.7) [52–85]	
Male gender	167 (61%)	
Education, years, mean (SD) [range]	12.5 (3.8) [7–25]	
Frailty index score, mean (SD)		0.23 (0.13)
Frailty index, categories ^a^		
Fit	51 (19%)	
Vulnerable	63 (24%)	
Mild frailty	61 (23%)	
Moderate frailty	51 (19%)	
Severe frailty	36 (14%)	
Frailty domain: cardiovascular morbidity		0.16 (0.14)
Angina (*n* = 275)	14 (5%)	
Atrial fibrillation/flutter	30 (11%)	
Heart failure	7 (3%)	
Coronary heart disease (*n* = 275)	50 (18%)	
Diabetes mellitus	10 (4%)	
Hypertension	152 (55%)	
Myocardial infarction	32 (12%)	
Peripheral arterial disease	10 (4%)	
Previous stroke or transient ischemic attack	34 (12%)	
Frailty domain: non-cardiovascular morbidity		0.17 (0.12)
Anxiety	17 (6%)	
Arthritis	36 (13%)	
Asthma	17 (6%)	
Cancer diagnosed last 5 years	17 (6%)	
Chronic renal failure, GFR < 60ml/h	57 (21%)	
Chronic obstructive pulmonary disease (*n* = 275)	19 (7%)	
Degenerative back (sciatica or spinal stenosis) (*n* = 274)	30 (11%)	
Depression (*n* = 274)	54 (20%)	
Falls last year (*n* = 256)	182 (66%)	
Frailty domain: use of more than 5 medications (*n* = 274) ^b^	144 (53%)	
Frailty domain: need for help in personal ADL		0.16 (0.23)
Eating (*n* = 275)	11 (4%)	
Dressing and undressing (*n* = 275)	43 (16%)	
Grooming (*n* = 275)	50 (18%)	
Assistance or a walker for mobility (*n* = 259)	112 (41%)	
Getting in and out of bed (*n* = 273)	5 (2%)	
Bathing or showering (*n* = 273)	51 (18%)	
Restroom visits (*n* = 273)	5 (2%)	
Frailty domain: Need for help in instrumental ADL		0.43 (0.38)
Telephone (*n* = 274)	46 (17%)	
Transportation (*n* = 273)	105 (38%)	
Shopping	146 (53%)	
Cooking	144 (52%)	
Household chores (*n* = 275)	164 (59%)	
Managing own medications (*n* = 275)	91 (33%)	
Handling money or paying bills (*n* = 274)	114 (41%)	
Frailty domain: nutritional status ^b^		
Serum albumin < 35 g/dL (*n* = 274)	1 (0%)	
BMI < 21 kg/m2 (*n* = 243)	20 (8%)	
Frailty domain: Cognitive function		0.26 (0.33)
MMSE-NR categories, mean (SD):		
27–30 points	143 (52%)	
24–26 points	69 (25%)	
21–23 points	43 (16%)	
<21 points	21 (8%)	
Frailty domain: physical function		0.63 (0.36)
Gait speed, categories, mean (SD):		
≥1 m/s (*n* = 32)	32 (14%)	
0.80–0.99 m/s (*n* = 49)	49 (21%)	
0.60–0.79 m/s (*n* = 61)	61 (27%)	
<0.60 m/s (*n* = 87)	87 (38%)	
Descriptive data for variables analyzed as categorical in the FI		
Serum albumin, g/dL, mean (SD) [range]	44.8 (3.5) [33–78]	
BMI, kg/m^2^, mean (SD) [range] (*n* = 167)	27.6 (4.4) [18.4–41.8]	
Gait speed, m/s, mean (SD) [range] (*n* = 229)	0.70 (0.26) [0.07–1.43]	

Frailty was assessed by 37 items from the 48 item Frailty Index (score 0 or 1) as number of frailty indicators present divided by number assessed. For each domain of frailty, we similarly assessed the fraction (range 0–1) of positive frailty indicators within that particular domain. GFR, Glomerular filtration rate; BMI, Body Mass Index; ADL, Activities of daily living; MMSE-NR, Norwegian Revised Mini Mental State Evaluation.

a Frailty categorized as follows: Fit: FI < 0.10, Vulnerable: FI: 0.10–0.19, Mild degree of frailty: FI: 0.20–0.29. Moderate degree of frailty: FI: 0.30–0.39. Severe degree of frailty: FI ≥ 0.40.

b Included in Frailty Index score, mean (SD) score for this domain of the Frailty Index is not calculated.

### Cognitive profile

**Figure 2:**
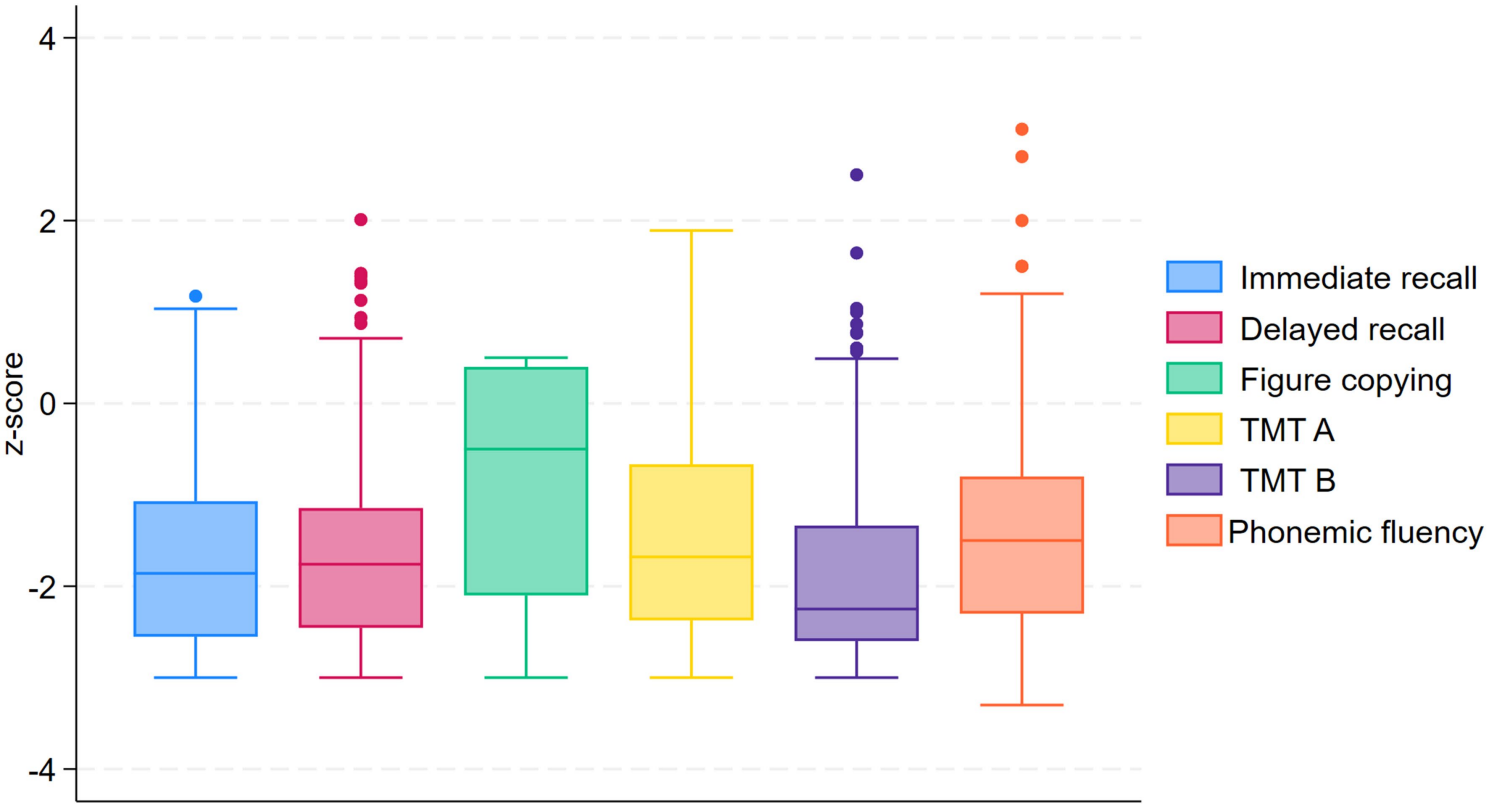
Box plot for different cognitive domains compared to normative data.

On average, the patients had reduced function in all cognitive domains compared to normative populations of the same age and length of education, as displayed in Table 2. The *z*-score distributions were similar across the different cognitive tests, with median *z*-scores around −1.6 and the 75-percentile around −1 as presented in Figure 2. The figure copying task represents an exception, as the mean *z*-score for this task was −0.85 and the 75-percentile above 0. The mean score on the Clinical Dementia Rating Scale was 0.68, which is between mild cognitive impairment and mild dementia, as shown in Table 3.

**Table 2:** Cognitive test results.

	*n*	Raw score			Mean Z-score (SD)^†^
		Mean (SD)	Median (IQR)	Range	
Screening tests					
MMSE-NR, points (out of 30)	276		27 (24–29)	15–30	
Clock Drawing Test, points (out of 5)	276		4 (3–5)		
Memory tests					
CERAD 10-word memory test ^a^					
Immediate recall, words	77	12 (3.9)	11 (9–14)	1–20	−1.95 (0.84)
Delayed recall, words	77	2 (1.8)	1 (0–3)	0–6	−1.60 (1.10)
RAVLT ^a^					
Immediate recall, words	199	29 (9.2)	28 (22–35)	11–57	−1.66 (1.03)
Delayed recall, words (trial 7)	196	4 (2.8)	4 (2–6)	0–12	−1.68 (0.97)
CERAD 10-word memory test and RAVLT merged ^a^					
Immediate recall	276				−1.74 (0.98)
Delayed recall	273				−1.66 (1.01)
CERAD figure					
Figure copying, points (out of 11)	264	10 (1.6)	10 (9–11)	2–11	−0.85 (1.35)
Trail making tests					
TMT A, sec ^b^	269	74 (39.8)	63 (47–88)	20–266	−1.50 (1.09)
TMT B, sec ^b^	180	183 (104.3)	161 (109–229)	44–840	−1.88 (1.03)
Fluency tests					
Phonemic fluency, number of words	246	25 (12.0)	25 (17–31)	1–79	−1.46 (1.10)
Semantic fluency animals, number of words ^c^	244	14 (5.7)	13 (10–18)	3–33	
Semantic fluency clothes, number of words ^c^	191	14 (5.9)	14 (10–17)	3–43	
Semantic animal + clothing ^c^	237				−1.59 (1.20)
IQCODE, mean score	231	3.62 (0.55)			

a For assessment of verbal memory, either CERAD 10-word test or RAVLT was applied. *Z*-scores were calculated for both, thus enabling a common measure for immediate and delayed recall across choice of verbal memory test.

b 4 were unable to complete TMT A, and 92 were unable to complete TMT B. Mean, SD, median, IQR and range are for those who completed, whereas *Z*-scores are estimated also for those who did not. For details, see text.

c For participants ≤77 years, *Z*-scores for semantic fluency was estimated for animals + clothes, whereas for those >78 years it was estimated for animals only. For details, see text.

^†^*Z*-scores estimated for those who completed.

IQR, Interquartile range; MMSE-NR, Norwegian Revised Mini Mental State Evaluation; CERAD, Consortium to Establish a Registry for Alzheimer’s Disease; RAVLT, Rey Auditory Verbal Learning Test; IQCODE, Informant Questionnaire on Cognitive Decline in the Elderly.

**Table 3:** Scores on the Clinical Dementia Rating Scale (CDR), algorithm based.

	n	0	0.5	1	2	3	Mean (SD)
CDR total score, n (%)	261	18 (6.9)	171 (65.5)	52 (19.9)	19 (7.3)	1 (0.4)	0.68 (0.47)
Memory, n (%)	270	28 (10.4)	141 (52 0.2)	89 (33.0)	12 (4.4)	0	0.68 (0.42)
Orientation, n (%)	269	161 (59.9)	76 (28.3)	26 (9.7)	6 (2.2)	0	0.28 (0.42)
Judgment and problem solving	269	85 (31.6)	103 (38.3)	63 (23.4)	15 (5.6)	3 (1.1)	0.57 (0.57)
Community affairs, n (%)	268	49 (18.3)	124 (46.3)	67 (25.0)	26 (9.7)	2 (0.8)	0.70 (0.58)
Home and leisure interests, n (%)	270	64 (23.7)	109 (40.4)	56 (20.7)	37 (13.7)	4 (1.5)	0.72 (0.68)
Personal care, n (%)	265	211 (79.6)	1 (0.4)	38 (14.3)	12 (4.5)	3 (1.1)	0.27 (0.60)

A score of 0 points signifies the absence dementia, 0.5 points mild cognitive impairment, 1 point mild dementia, 2 points moderate dementia, and 3 points severe dementia.

### Cluster analysis

The results of the cluster analysis are presented in Table 4. Of the included patients, 239 had complete data for immediate and delayed recall, figure copying, TMT A and B and phonemic verbal fluency and were included in this analysis. We explored models with two, three and four clusters, and found that a three-cluster model provided a clinically interpretable grouping of the participants. The K-means analysis distinguished the three clusters as described in Table 4. Cluster 1 included 51 patients (21.3%) with a relatively good performance on all cognitive tests and mean *z*-scores mostly between −0.1 and −0.7. We named this cluster “Mild symptoms.” Cluster 2 was the largest one, with 117 patients (49.0%). They had severe symptoms with mean *z*-scores varying between −1.5 and −2.2 with the exception that they performed nearly normally on figure copying task (mean *z*-score −0.05). We named this cluster “Severe symptoms, with preserved visuoconstructive ability.” Cluster 3, including 71 patients (29.7%), performed poorer on all tests, with mean *z*-scores varying between −1.9 and −2.5.

**Table 4:** Cluster analysis.

	Cluster 1 Mild symptoms	Cluster 2 Severe symptoms, with preserved visuospatial ability	Cluster 3 Severe symptoms	*p* (ANOVA)
	*n* = 51 (21.3%)	*n* = 117 (49.0%)	*n* = 71 (29.7%)	
Cognitive tests, mean (SD) Z-scores				
Immediate recall merged	−0.68 (0.99)	−1.86 (0.84)	−2.13 (0.75)	
Delayed recall merged	−0.72 (1.00)	−1.88 (0.82)	−1.89 (0.92)	
Figure copying	−0.14 (0.96)	−0.05 (0.63)	−2.51 (0.66)	
TMT A	−0.19 (0.95)	−1.51 (0.87)	−2.20 (0.74)	
TMT B	−0.36 (0.99)	−2.16 (0.70)	−2.37 (0.53)	
Phonemic fluency	−0.52 (1.15)	−1.53 (0.99)	−2.01 (0.80)	
Descriptive variables, mean (SD)				
Age	72 (6)	73 (5)	73 (6)	0.56
Education, years	13 (4)	13 (4)	12 (4)	0.26
MMSE, sum score	28 (2)	26 (3)	24 (4)	<0.01
CDT, sum score	5 (1)	4 (1)	3 (1)	<0.01
Physical function, mean (SD)				
TUG time	14.9 (6.4)	20.6 (16.8)	20.8 (9.1)	<0.05
SPPB total sum	8 (3)	6 (3)	4 (3)	<0.01
Total steps, 10 m	18 (4)	24 (11)	28 (12)	<0.01
iNPH gait	68.3 (23.3)	48.4 (25.8)	33.7 (22.7)	<0.01
iNPH balance	72.1 (17.0)	61.9 (21.1)	51.4 (21.5)	<0.01
Gait speed m/s	0.90 (0.22)	0.70 (0.24)	0.60 (0.72)	<0.01
Frailty index	0.14 (0.09)	0.23 (0.13)	0.29 (0.13)	<0.01
CDR score	0.4 (0.2)	0.7 (0.4)	0.8 (0.6)	<0.01

### Physical assessment

Table 5 shows physical test results. The patients performed poorly on all the tests, with a mean SPPB score of 6.0 and 84% having a gait speed less than 1.0 m/s. Walking aids were used by 41% of the participants.

**Table 5:** Physical test results.

	*n*	Mean (SD)	Median (IQR)
TUG, seconds	210	19.7 (13.2)	15.8 (12.5–22.8)
SPPB total score, points (out of 12)	255	6.0 (3.5)	6 (3–9)
Balance, points (out of 4)	256	2.1 (1.5)	2 (1–3)
4 m gait, points (out of 4)	255	2.5 (1.3)	3 (1–4)
5 rises, points (out of 4)	256	1.3 (1.3)	1 (0–2)
10 meters walking			
Speed, m/s	246	0.77 (0.28)	0.77 (0.57–0.98)
Total steps	247	24.1 (10.4)	21 (17–27)
iNPH scale, gait, points	256	46.9 (27.4)	43.3 (23.7–70.3)
iNPH scale, balance, points	256	59.6 (22.3)	67 (50–67)

SPPB, Short Physical Performance Battery, higher score indicates better performance; TUG, Timed up and Go; iNPH scale, higher score indicates better performance.

### Frailty

The mean (SD) FI score was 0.23 (0.13), indicating mild degree of frailty, though with marked differences between the individual frailty domains. Physical function (gait speed) was most often affected (mean domain index 0.63) followed by need for help in iADL (mean domain index 0.43). The frailty domain polypharmacy comprises only one binary indicator (use of more than 5 medications), and for 53% of the patients this indicator was present. Of the two binary malnutrition indicators, less than 1% had serum albumin below 35 g/dL, whereas 8% had Body Mass Index (BMI) < 21 kg/m^2^. For the remaining frailty domains, the domain indexes were between 0.1 and 0.3 (Table 1).

## Discussion

Our main finding was that iNPH patients compared with normative data presented no specific cognitive profile; they had reduced cognitive function across all domains, though with considerable heterogeneity regarding degree of visuoconstructive impairment. Most iNPH patients lived with mild frailty, but the degree of frailty differed considerably between domains and was very high for physical function. This observation holds promise, since physical function is the symptom that is most likely to improve by shunt surgery (35).

### Cognitive function

Our patients demonstrated marked cognitive impairments on all tests. Mean *z*-scores varied roughly from −1.5 to −2, except from figure copying with a mean *z*-score of −0.85. The standard deviations of the *z*-scores were around 1 for most of the cognitive testes, which is per definition the same as in the normative population, indicating that the variance among the iNPH patients are similar to that among healthy individuals.

Cluster analysis suggested that three groups can be described regarding cognitive function. Cluster 1 exhibited mild symptoms with mean *z*-scores between −0.7 and −0.1. They were also less frail and had milder physical impairments than the two other clusters. We find cluster 2 to be of particular interest, comprising almost 50% of the participants. They had severe cognitive symptoms, very similar to cluster 3, with the exception that they performed nearly normally regarding figure copying, indicating a better preserved visuosconstructive function. Regarding frailty and physical function, their performances were intermediate between the two other clusters. Cluster 3 had more severe cognitive symptoms with *z*-scores between −1.9 and −2.5 for all the cognitive tests, including figure copying, indicating a global cerebral dysfunction. They were also more frail and had more severe physical impairments. Cluster analyses are indeed explorative and should be interpreted with caution, but in our opinion this finding warrants further and perhaps confirmatory studies.

When cognitive profile in iNPH is described, a frontal lobe dysfunction, with reduced psychomotor speed, attention and executive dysfunction is often emphasized in the early phase, followed by a global cognitive impairment as the disease progresses (36–38). None of the clusters we describe were characterized by typical frontal lobe dysfunction. Neither could we confirm the finding that memory might be the least affected domain in iNPH (39–41). Immediate as well as delayed verbal recall were reduced in all three clusters. This supports the notion that there is no specific cognitive profile among NPH patients, but rather that their cognitive performance is globally reduced, with severity increasing over time. Our findings align with other studies demonstrating that iNPH patients have reduced cognitive function across several rather than one specific cognitive domain (4, 42–46). Bluett et al. reviewed 81 articles and did not identify a specific cognitive profile (42). The choice of cognitive tests varied, however, significantly across studies. In a multicenter study, Hellström et al. included 142 iNPH patients who performed significantly weaker compared to healthy controls on all cognitive tests (43). Picascia et al., in their review, highlight that iNPH is a complex syndrome and that the pathology is not fully understood, reasonably explaining the variety in cognitive profiles reported (4).

Our patients had generally better CDR scores than what the cognitive test would suggest. This is particularly evident in cluster 2 and 3, where the average *z*-scores were around −2, typically indicating moderate dementia. Their mean CDR scores were 0.7 and 0.8, respectively, which correspond to levels between mild cognitive impairment and mild dementia.

### Frailty

Most patients in our material lived with mild frailty. Physical function was the most severely affected frailty domain, followed by need for help in iADL. The ADL impairments were most pronounced in activities that depend on mobility, such as household chores, shopping and cooking, with more than 50% of individuals affected. Functions often requiring higher cognitive abilities, such as managing own medications, were less commonly affected, with only 33% of individuals needing assistance. The burden of comorbidities was low, except for hypertension, which is in line with other studies (47). Regarding pADL, the same pattern emerged, with 41% using walking aids contributing to an elevated frailty index, but few other impairments were seen. The nutritional status of the patients was good, but polypharmacy was frequent with more than half the patients using more than five medications. If the frailty profile had been dominated by other factors, such as comorbidity or malnutrition, the symptoms might be expected to be less reversible. A cumulative deficits model of frailty has been shown to be useful in clinical medicine, and is an important risk factor for complications, death, and functional loss after surgery (48). The calculation of indexes for individual subdomains of FI is not validated, so these indexes must be interpreted with caution. In our opinion, however, a more pronounced insight into the frailty consequences of NPH might be provided through this approach. Some aspects of frailty may improve by successful treatment of iNPH, e.g., mobility and cognitive problems, while others may be unaffected and, on the contrary, deflate the effect of iNPH treatment and increase the risk of complications. Longitudinal studies are necessary to assess whether frailty profile can predict shunt response and to which degree frailty is reversible in iNPH patients.

### Clinical impact

We did not identify a specific cognitive profile typical for iNPH. The cognitive profile in this patient group seems to be heterogeneous without a particularly identifying pattern. Thus, cognitive assessment may be of limited use in diagnosing iNPH. We argue that it is nevertheless valuable to describe the cognitive symptoms sufficiently comprehensively in order to assist the patients with their complex needs. We recommend assessing memory, executive function, attention, and visuoconstructive function to ensure that potentially important impairments are detected. Which cognitive domains that are most prone to improve as a result of shunt surgery and whether the cognitive profile can be used to identify patients most likely to be shunt responders, must be assessed in longitudinal studies. Regarding frailty, our results indicate that mobility is in particular influenced by iNPH. Pronounced frailty in other domains might be the result of other processes that must be addressed by other measures than shunt surgery and, in the worst case, indicate a poorer prognosis and a higher risk of postoperative complications. Thus, a better understanding of different aspects of frailty in iNPH patients may help to improve treatment strategies. A future research priority will be to study how frailty responds to shunt surgery.

### iNPH

All included patients fulfilled the American-European criteria of possible iNPH (2) and constitute a clearly defined group, which strengthens generalizability. A caveat is that the current guidelines are debated (49); as such the American-European and Japanese iNPH guidelines differ somewhat (1, 2). It may therefore be discussed who are “true” iNPH patients; in this regard the category “Definite iNPH” according to the Japanese guidelines (1), refer to iNPH patients that respond favorably to shunt surgery. With the preoperative work-up of iNPH at this hospital, a high success rate for surgical response to shunt surgery has previously been reported (50).

### Strengths and limitations

Notable strengths in this study are the large patient population, the use of a comprehensive cognitive test battery with available comparison to population norms, and the simultaneous assessment of cognition, physical function, and frailty. The use of two different tests for verbal memory might constitute a possible source of error, but the use of *z*-scores based on large and relevant normative populations presumably makes it reasonable to use the two methods interchangeably. In the CERAD figure copying test, half of the norm material achieved a full score, indicating a ceiling effect and thus a reduced ability to discriminate in the upper end of the performance spectrum. About 30% of those accepted for surgery were not included in our material. Even if this mostly was due to practical reasons such as, vacations, capacity in the research team etc., we cannot entirely exclude some degree of selection bias. Assessment of the relationship between cognitive test profile on one hand and radiological features and CSF biomarkers on the other, as described by other authors (51, 52), might have strengthened the results. Finally, it was beyond the scope of this study to assess how the studied variables associate with clinical response to the only available treatment for iNPH, i.e., CSF diversion (shunt) surgery. This has to be assessed in separate studies.

## Conclusion

We found a non-specific and general cognitive impairment in iNPH, indicative of a general cerebral affection in these patients. iNPH patients are a heterogeneous group with varying degree of cognitive impairment. A comprehensive cognitive assessment will be of limited use in diagnosing iNPH but may be useful for alleviating the complex needs for this patient group. We need more studies on how frailty assessment can be used to improve treatment decisions in patients with suspected iNPH, possibly contributing to a more tailored and individualized treatment.

## Data Availability

The raw data supporting the conclusions of this article will be made available by the authors, without undue reservation.
