# Correction: Cognitive profile and frailty in patients with idiopathic normal pressure hydrocephalus

**DOI:** 10.3389/fneur.2025.1708210

**Published:** 2025-10-13

**Authors:** Magnhild S. Dejgaard, Per Kristian Eide, Gro Gujord Tangen, Eva Skovlund, Geir Selbæk, Torgeir Bruun Wyller

**Affiliations:** ^1^Department of Geriatric Medicine, Oslo University Hospital, Oslo, Norway; ^2^Institute of Clinical Medicine, University of Oslo, Oslo, Norway; ^3^Department of Neurosurgery, Oslo University Hospital, Oslo, Norway; ^4^KG Jebsen Centre for Brain Fluid Research, Oslo, Norway; ^5^Norwegian National Center for Ageing and Health, Vestfold Hospital Trust, Tønsberg, Norway; ^6^Department of Public Health and Nursing, Norwegian University of Science and Technology (NTNU), Trondheim, Norway

**Keywords:** normal pressure hydrocephalus, gait disorder, dementia, frailty, mild cognitive impairment

The title of this article was erroneously given as: [**Cognitive profile and frailty in patients with idiopathic normal pressure**]. The correct title of the article is [**Cognitive profile and frailty in patients with idiopathic normal pressure hydrocephalus**].

The original version of this article has been updated.

